# STENSL: Microbial Source Tracking with ENvironment SeLection

**DOI:** 10.1128/msystems.00995-21

**Published:** 2022-09-01

**Authors:** Ulzee An, Liat Shenhav, Christine A. Olson, Elaine Y. Hsiao, Eran Halperin, Sriram Sankararaman

**Affiliations:** a Department of Computer Science, University of California Los Angeles, Los Angeles, California, USA; b Center for Studies in Physics and Biology, Rockefeller University, New York, New York, USA; c Department of Integrative Biology and Physiology, University of California Los Angeles, Los Angeles, California, USA; d Department of Computational Medicine, University of California, Los Angeles, California, USA; e Department of Human Genetics, University of California, Los Angeles, California, USA; f Department of Anesthesiology and Perioperative Medicine, University of California, Los Angeles, California, USA; Michigan State University

**Keywords:** feature selection, microbial source tracking, microbiome, mixture models, sparsity

## Abstract

Microbial source tracking analysis has emerged as a widespread technique for characterizing the properties of complex microbial communities. However, this analysis is currently limited to source environments sampled in a specific study. In order to expand the scope beyond one single study and allow the exploration of source environments using large databases and repositories, such as the Earth Microbiome Project, a source selection procedure is required. Such a procedure will allow differentiating between contributing environments and nuisance ones when the number of potential sources considered is high. Here, we introduce STENSL (microbial Source Tracking with ENvironment SeLection), a machine learning method that extends common microbial source tracking analysis by performing an unsupervised source selection and enabling sparse identification of latent source environments. By incorporating sparsity into the estimation of potential source environments, STENSL improves the accuracy of true source contribution, while significantly reducing the noise introduced by noncontributing ones. We therefore anticipate that source selection will augment microbial source tracking analyses, enabling exploration of multiple source environments from publicly available repositories while maintaining high accuracy of the statistical inference.

**IMPORTANCE** Microbial source tracking is a powerful tool to characterize the properties of complex microbial communities. However, this analysis is currently limited to source environments sampled in a specific study. In many applications there is a clear need to consider source selection over a large array of microbial environments, external to the study. To this end, we developed STENSL (microbial Source Tracking with ENvironment SeLection), an expectation-maximization algorithm with sparsity that enables the identification of contributing sources among a large set of potential microbial environments. With the unprecedented expansion of microbiome data repositories such as the Earth Microbiome Project, recording over 200,000 samples from more than 50 types of categorized environments, STENSL takes the first steps in performing automated source exploration and selection. STENSL is significantly more accurate in identifying the contributing sources as well as the unknown source, even when considering hundreds of potential source environments, settings in which state-of-the-art microbial source tracking methods add considerable error.

## INTRODUCTION

Complex microbial communities are present in multiple biological domains and play far-reaching roles in various fields, from human health, through agriculture, to bioremediation. The study of these high-dimensional communities offers great opportunities for biological discovery, due to the ease of their measurement, the ability to perturb them, and their dynamic and rapidly evolving nature. These same characteristics, however, make it difficult to extract informative and reproducible patterns informing the origins of these ecosystems. Specifically, as microbial community assembly strongly depends on the dispersal of microbes from a mixture of source environments, the analysis of such communities requires tailored algorithms deconvolving latent structures regarding community integration.

Over the last decade, several computational techniques have been proposed for tracking the assembly of such complex microbial communities (1–3). By performing “microbial source tracking,” methods such as FEAST (1) and SourceTracker2 (2) quantify the fraction, or proportion, of different microbial samples (sources) in a target microbial community (sink), while assuming the sink is a mixture of sampled microbial environments (i.e., known sources) with the possibility of unmeasured ones, collectively referred to as the “unknown source.” These methods have shown great promise in revealing new insights, particularly in quantifying contamination and tracking microbial community integration (4–6). However, in many practical scenarios the number of contributing sources is much smaller than the number of candidate sources considered in the analysis. Unfortunately, existing methods are suboptimal in such scenarios, hindering the concept of source exploration.

As it may be nearly impossible to obtain sequencing data for all potential source environments in a study, source exploration using public repositories may augment microbial source tracking analyses, beyond the scope of any one study. We therefore suggest that in these settings, microbial source tracking can benefit from automated source exploration and selection. Nonetheless, this process remains largely understudied, with current methods not suitable for the task, as-is, since the estimation error increases as the number of sources considered increases. Only one previous study tried to address this limitation by exploring the utility of Aitchison distance to select “important” sources that drive community assembly (7). However, as we demonstrate using simulations, the accuracy of this strategy in the presence of an unknown source is very low.

Here we introduce STENSL, a scalable algorithm that unveils the latent structure of a given microbial community by modeling it as a convex combination of (1) contributing sources (observed sources with a nonzero contribution to the sink), (2) nuisance or noncontributing sources (observed sources with zero contribution), and (3) unobserved or unknown sources. We use the term candidate sources to describe the union of the former two. STENSL enables the incorporation of multiple candidate sources from publicly available repositories without the need for manual selection. Unlike current microbial source tracking methods, multiple sources can be considered without increasing the error in estimating the underlying mixing proportions. We demonstrate that, when considering both contributing and noncontributing source environments, STENSL is significantly more accurate than state-of-the-art methods. Thus, by leveraging sources from publicly available repositories, STENSL can provide more accurate estimates of the origin of complex microbial communities.

## RESULTS

### A brief description of the model.

**FIG 1:**
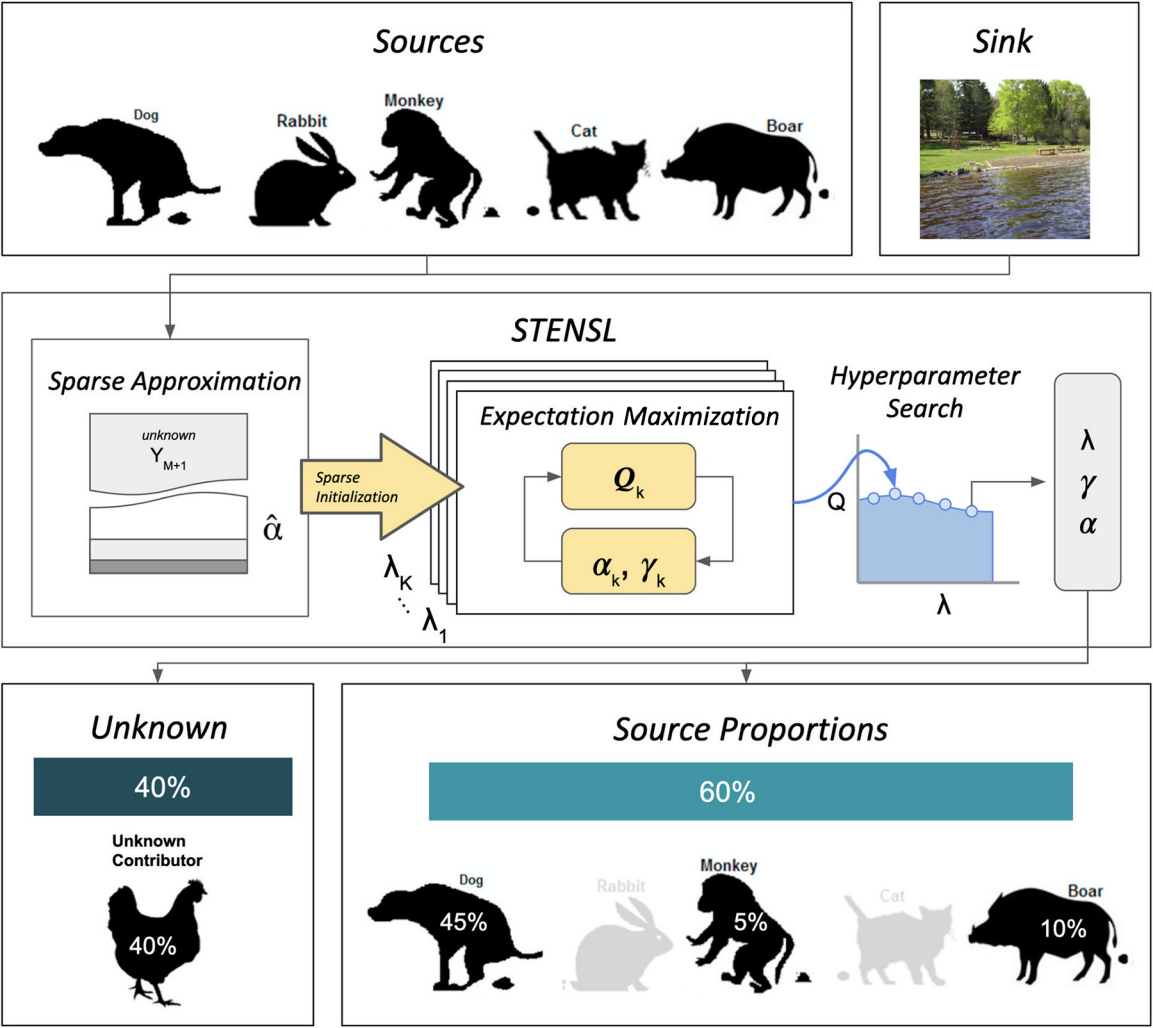
Overview of the STENSL algorithm. The source-tracking task involves estimation of the relative contribution of sources to the formation of a sink. STENSL performs sparse estimation which makes source tracking more accurate for large numbers of sources and allows consideration of sources that may not contribute to the sink.

STENSL detects a core group of source environments within a larger group of candidate environments and quantifies their contribution to the formation of complex microbial communities. STENSL takes an input a microbiome sample (called the sink) as well as a separate group of microbial samples (called the candidate sources), detects a core group of contributing sources and estimates the fraction of the sink community that was contributed by each of these core environments. By virtue of these mixing proportions summing to less than the entire sink, STENSL also reports the fraction of the sink attributed to other, unobserved, origins (Fig. 1). STENSL is based on a least-squares optimization with an L1-norm regularization, acting as a source selection procedure, integrated into the microbial source tracking mixture model. In STENSL, we also introduce a procedure to analytically reconstruct the best representation of the unknown source. Specifically, we leverage the regularized least-squares solution of the mixture model to identify taxa which are accurately reconstructed and are thus not unlikely to originate from the unknown source (Materials and Methods). STENSL identifies three types of sources: (1) contributing sources (sources observed and having a nonzero contribution), (2) nuisance sources, also called noncontributing sources (observed and having zero contribution), and (3) unobserved sources, collectively referred to as the unknown source. In other words, our method explicitly differentiates between two types of known sources introduced into the model (i.e., contributing and nuisance). As we demonstrate below, these modifications are significant in denoising and exploring a cohort of candidate sources. Specifically, we show that STENSL is significantly more accurate than existing methods when considering a large number of nuisance sources, a setting in which the identification of truly contributing sources becomes nontrivial.

### Model evaluation using data-driven simulations.

**FIG 2:**
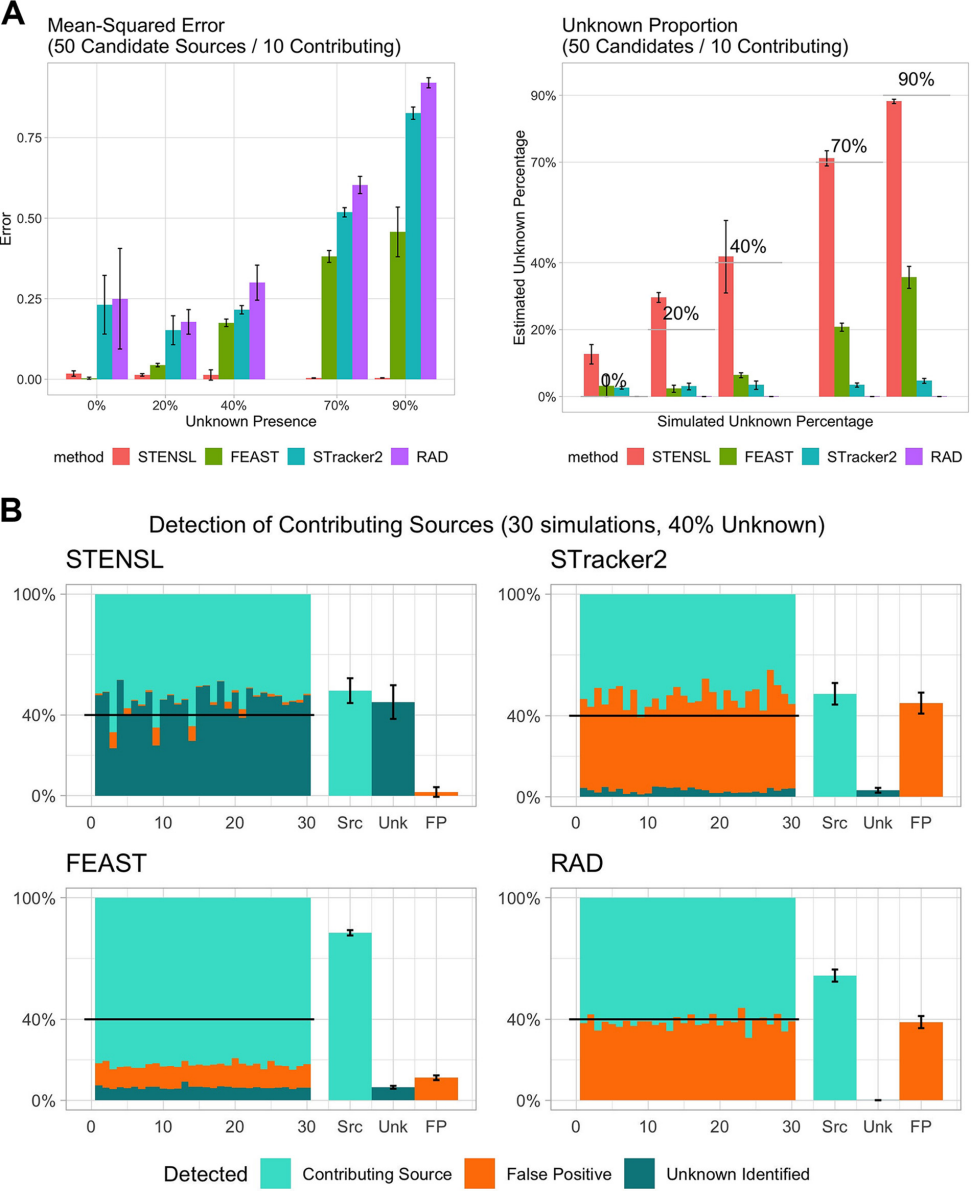
(A) Estimation of mixing proportions using STENSL. In a simulated setting of M = 50 total candidates (K = 10 contributing sources within), mean-squared error of the estimated mixing proportion was evaluated for STENSL, SourceTracker2, FEAST, and RAD. The accuracy of estimating the simulated unknown proportion was also measured using absolute error. Experiments were repeated with increasing unknown presence of 0%, 20%, 40%, 70%, and 90%. Error bars indicate standard deviation in error for tested sinks within each group. (B) Breakdown of estimated mixing proportions. For the group of sinks where we simulated intermediate unknown proportion (40%), we label how each method attributes mixing proportion weights across truly contributing sources, false positive sources, and the unknown proportion, in the setting of 50 candidate and 10 contributing sources. STENSL maintained the lowest proportion of false positive attributions in comparison to similar methods which were overwhelmed adversely by the increased number of candidates (significantly lower with *P* < 7.47 × 10^−4^ across all methods, Fig. S1).

We use simulations to compare the accuracy of STENSL to FEAST, SourceTracker2, and RAD, methods previously suggested for microbial source tracking. The samples used in these simulations are based on real microbial samples documented and processed as part of the Earth Microbiome Project (8). The synthetic sink samples were generated as a convex combination of real microbial samples (i.e., contributing sources) and an unknown source, hidden from the algorithm. Our evaluation extends the source tracking problem by introducing to the algorithm numerous additional sources unrelated to the sink such that all methods consider both contributing and nuisance sources. To measure accuracy, we compared the estimated mixing proportions with the true ones using mean-squared error (Figure 2A, Table S1). Overall, we found that STENSL was the only method to consistently estimate the level of real sources’ contribution, with significantly lower mean-squared error (MSE) across positive unknown contributions up to 90% (SourceTracker2, *P* < 9.13 × 10^−7^; FEAST, *P* < 1.01 × 10^−6^; RAD, *P* < 9.13 × 10^−7^; Wilcoxon signed-rank test). The ability of each method to distinguish truly contributing sources was further assessed by summing non-zero weight attributed to nuisance sources. We term this metric the “false positive rate” (see Materials and Methods).

10.1128/mSystems.00995-21.7 TABLE S1 Performance table benchmarking methods. (a) *P*-values for difference in mean t-tests (paired) for MSE accuracy of STENSL to estimate the true mixing proportions against comparable methods. We observe that for any amount of positive unknown contribution that STENSL would obtain favorable results in the estimation task with significance. (b) *P*-values for difference in mean t-tests of false positive source detections, characterized as noisy predictions. (c) *P*-values for difference in mean t-tests of the absolute error between simulated and estimated unknown presence. Download Table S1, DOCX file, 0.02 MB. Copyright © 2022 An et al. 2022 An et al. https://creativecommons.org/licenses/by/4.0/ This content is distributed under the terms of the Creative Commons Attribution 4.0 International license.

We found that using STENSL, the false positive rate was significantly reduced compared to the other methods (Figure 2B; SourceTracker2, *P* < $9.13\times 10^{-7}$; FEAST, *P* < 1.67 × 10^−6^; RAD, *P* < $9.13\times 10^{-7}$; Wilcoxon ranked-sum test). We note that lower false positive rates correspond to improvements in the identification of truly contributing sources as well as estimation of the unknown source proportions. Conversely, we found that for FEAST, RAD, and SourceTracker2, noncontributing sources were consistently assigned positive weights and the misattributions increased with an increasing number of candidate sources. Notably, in the absence of nuisance sources, STENSL is as accurate as FEAST, the state-of-the-art method.

10.1128/mSystems.00995-21.1 FIG S1 Performance of STENSL, FEAST, SourceTracker2, and RAD where no unknown source is present (0%) in simulations. Estimation performance is measured on a simulated sink consisting of K = 10 sources, with M = 50 sources provided in total as potential candidates as sources in the source tracking problem. For 30 such simulated source-sink problems (a) performance is quantified as mean-squared error of known simulated mixing proportion vs estimated mixing proportion and (b) sum of residuals (absolute) of estimated mixing proportion for each set of sources where error directly corresponds to misattributed proportions. Download FIG S1, TIF file, 0.8 MB. Copyright © 2022 An et al. 2022 An et al. https://creativecommons.org/licenses/by/4.0/ This content is distributed under the terms of the Creative Commons Attribution 4.0 International license.

We next quantified the accuracy of the estimated unknown proportion through absolute error (AE) against the true simulated unknown proportion ranging from 0% to 90% in the sink. To visualize how unknown proportions affect estimation, we correlated the estimated unknown proportions with the true unknown proportions in Fig. 2A. We found that STENSL is significantly more accurate in estimating the unknown source contribution whenever there was positive unknown presence (SourceTracker2, *P* < 2.04 × 10^−6^; FEAST, *P* < 1.63 × 10^−5^; RAD, *P* < 9.13 × 10^−7^; Wilcoxon rank-sum test; Table S1). For the smallest simulated setting of three true sources and six total candidate sources, we found that all methods were accurate in estimating the unknown source contribution (Fig. S2). However, as the number of candidate sources increased, existing methods significantly underestimated the unknown proportion due to both false identifications of sources and overestimation of truly contributing sources. In addition to stimulated sinks, we also examined the effectiveness of STENSL in discerning sources with commonality in descriptive features from the Earth Microbiome Project (EMP). We gathered samples as candidates from up to 10 separate EMP studies and found that STENSL estimated proportions highly for sources from the study where the sink was originally found (Fig. S6).

10.1128/mSystems.00995-21.2 FIG S2 Performance of source tracking algorithms using a small number of sources. K = 3 contributing sources were present among M = 6 candidate sources with 40% unknown contribution. 5 total simulations are visualized. Estimated source proportions were colorized as being correctly attributed to true sources (Src), false-positive detection (FP), attributed correctly as unknown proportion (Unk), and overly attributed to a source despite being correct (Over). Download FIG S2, TIF file, 0.5 MB. Copyright © 2022 An et al. 2022 An et al. https://creativecommons.org/licenses/by/4.0/ This content is distributed under the terms of the Creative Commons Attribution 4.0 International license.

10.1128/mSystems.00995-21.6 FIG S6 Earth Microbiome Project experiments. (a) A single sea water sample was chosen from EMP study 1222 (S5), and its source proportion was estimated given samples collected across 8 total separate studies including the originating study. Up to 100 samples were considered from each study. (b) A similar experiment was performed for an urban biome sample chosen from EMP study 2192 (S10), given other samples spanning 10 separate studies. The estimated proportions were colorized by their origin; either the original study (Original) a separate study (External), or an unaccounted unknown proportion (Unknown). Download FIG S6, TIF file, 0.5 MB. Copyright © 2022 An et al. 2022 An et al. https://creativecommons.org/licenses/by/4.0/ This content is distributed under the terms of the Creative Commons Attribution 4.0 International license.

### *In vitro* model validation.

**FIG 3:**
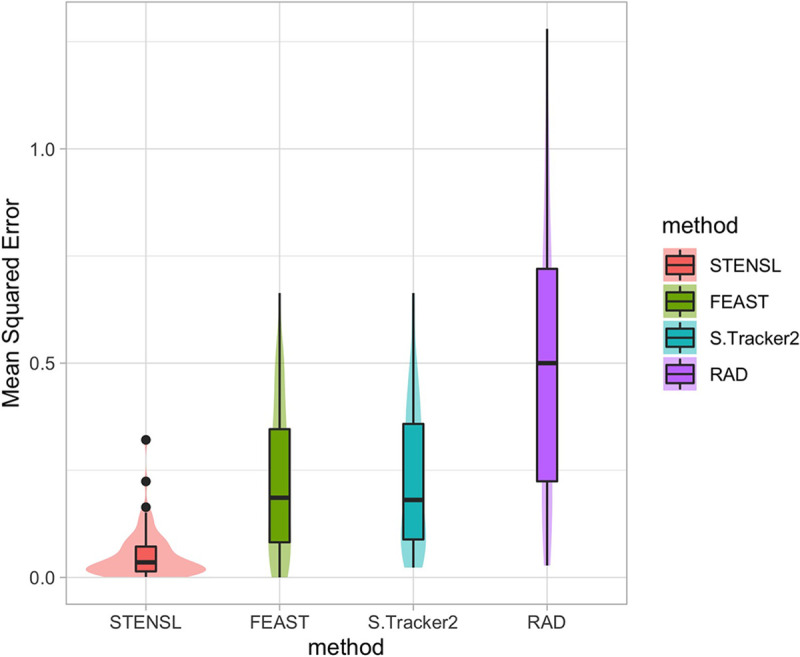
Analysis of *in vitro* data set of sinks created from mixture of human and mice gut samples. The accuracy of STENSL, SourceTracker2, and FEAST was evaluated using mean-squared error (MSE) against the true mixing proportion used to create *in vitro* sinks with unknown proportions ranging from 0% to ~80% and in the presence of 50 noncontributing sources.

To validate the sparsity assumption introduced by STENSL, which models a microbial community as a convex combination of contributing sources, and to further demonstrate the utility of our method, we created an *in vitro* data set. In this data, we generated *in vitro* sinks as a mixture of microbial samples acquired from the digestive systems of three human subjects and three mice subjects (Materials and Methods). Twenty-four *in vitro* sinks were assembled, each sink consisting of two to three microbial samples at varying mixing proportions. When performing microbial source tracking analyses on this set of contributing sources and sinks, we added a group of 50 additional noncontributing sources (Materials and Methods). Our analysis considered STENSL, SourceTracker2, FEAST, and RAD. We evaluated the accuracy of these methods by using MSE between the lab-generated ground-truth and the estimated mixing proportions. As part of our analysis, for each sink, we withheld one or two contributing sources to create settings in which the unknown proportions range between 0% to ~80%. Similar to our simulation results, we found that in real data, STENSL was significantly more accurate than other methods (Fig. 3; SourceTracker2, *P* < 2.2 × 10^−16^; FEAST, *P* < 2.1 × 10^−16^; RAD, *P* < 2.8 × 10^−16^; Wilcoxon ranked-sum test). The breakdown of estimated source proportions for a subset of sink samples are visualized in Fig. S5.

10.1128/mSystems.00995-21.5 FIG S5 Visualization of the *in-vitro* validation experiment using human and mice gut samples. 12 samples were mixed each containing one or two contributing sources and 30% unknown contribution. Source proportions were estimated in the presence of 50 candidate sources. Estimated source proportions were colorized as being correctly attributed to true sources (Src), false-positive detection (FP), attributed correctly as unknown proportion (Unk), and overly attributed to a source despite being correct (Over). Download FIG S5, TIF file, 0.5 MB. Copyright © 2022 An et al. 2022 An et al. https://creativecommons.org/licenses/by/4.0/ This content is distributed under the terms of the Creative Commons Attribution 4.0 International license.

**FIG 4:**
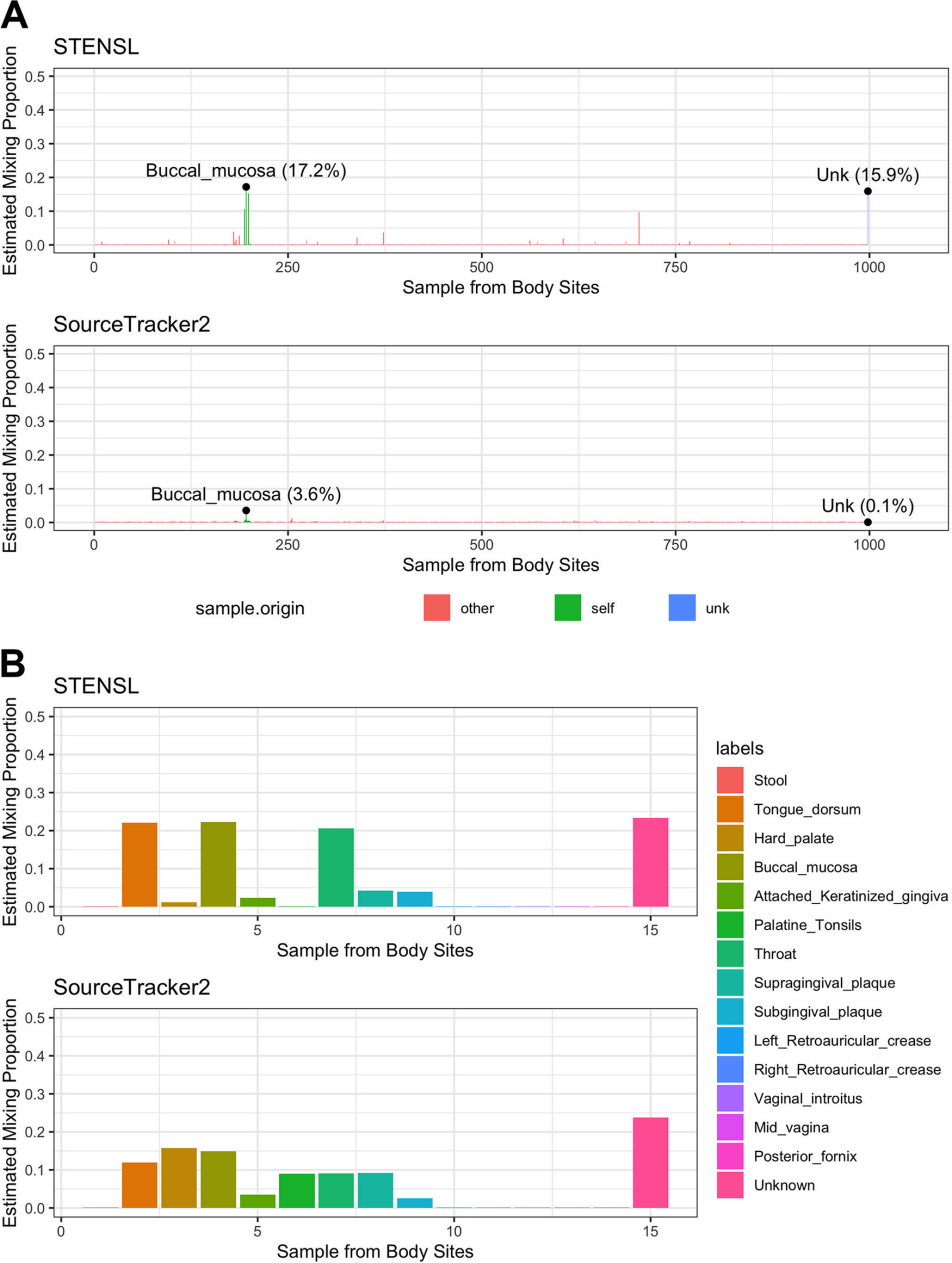
A single microbial sample can be strongly attributed to its original subject in a database-wide analysis of the Human Microbiome Project using STENSL. STENSL is applied in analyzing a saliva sample from one human subject as a composition of any choice of 997 samples which were collected from other body sites from the same subject and from other human subjects. (A) Using STENSL, we identify several samples with high contributions which originate from the same human subject. (B) To verify the database-wide analysis, we compared it with the results of applying both methods to the set of 15 sources belonging to the original human subject and no additional candidate sources from other human subjects.

**Source selection in the Human Microbiome Project.** To demonstrate the utility of STENSL when using large public repositories, we sought to assess the origins of a microbiome sample taken from a single individual found in the Human Microbiome Project (9). To construct the source selection problem, we defined the sink to be a saliva microbiome sample, which is one of several orally acquired samples including tongue, palate, and buccal mucosa. The candidate sources were then defined to be all the microbiome samples from the focal subject from which saliva was sampled (excluding saliva) as well as all other available microbial samples originating from 15 body sites across 88 individuals. In Fig. 4A, we outline results of applying both STENSL and SourceTracker2 to the same source tracking problem, with a substantially higher false positive rate attributed to the latter. Specifically, STENSL attributed a total of 43.1% to other oral microbiome samples belonging to the focal subject from which the sink was sampled (17.2% from buccal mucosa, 15.3% from tongue dorsum, and 10.6% from throat), while SourceTracker2 attributed only 4.9% to the other oral microbiome samples belonging to the focal subject from which the sink was sampled (3.6% from buccal mucosa, 0.6% from tongue dorsum, and 0.7% from throat). In addition, STENSL estimated an unknown contribution of 26% while estimating zero contribution for most noncontributing sources originating from other individuals. In contrast, SourceTracker2 assigned nonzero weights to the majority of sources from all other individuals (i.e., nuisance sources) and estimated a negligible unknown proportion (0.1%). We next performed a follow-up analysis, only considering samples from the focal individual (1 individual, 15 samples across body sites) and examined the results of this problem with no external individuals, a setting with little to no nuisance sources. Both methods estimated that the oral sites would contribute largely to the saliva (STENSL estimated 22.3% buccal mucosa, 22.1% tongue dorsum, and 20.5% throat; SourceTracker2 estimated 14.9% buccal mucosa, 12.0% tongue dorsum, and 9.1% throat), and an unknown proportion of approximately 20% (STENSL estimated 23.4% unknown, while SourceTracker2 estimated 23.7% unknown). These estimates agreed best with the results of STENSL in the full setting of 88 individuals and 997 samples. STENSL specifically highlighted tongue, buccal mucosa, and throat samples as the top contributors, which remained consistent when analyzing one subject or including the entire cohort. In addition to SourceTracker2, we also evaluated FEAST and RAD estimates on the HMP data, where we continued to observe little to no detection of the saliva samples and no identification of unknown presence (Fig. S4).

10.1128/mSystems.00995-21.4 FIG S4 Database-wide analysis of Human Microbiome Project using FEAST and RAD. We applied FEAST and RAD in estimating the contributors to a saliva sample in the Human Microbiome Project (HMP) given an array of candidate source samples originating from the individual and other individuals. Both FEAST and RAD estimated no unknown proportion (0%) which disagreed with initial findings using hand-selected samples from the individual as sources where unknown presence was estimated to be > 10%. Using FEAST, the top contributing source was estimated as Buccal Mucosa which was correctly attributed to the original individual, but only 7.5% of the sink was attributed. RAD did not attribute any of the provided sources from the original individual to the sink, identifying 14.9% of another individual’s sample in the sink. Download FIG S4, TIF file, 0.5 MB. Copyright © 2022 An et al. 2022 An et al. https://creativecommons.org/licenses/by/4.0/ This content is distributed under the terms of the Creative Commons Attribution 4.0 International license.

## DISCUSSION

In this work we present STENSL, an expectation-maximization algorithm with sparsity that enables the identification of contributing sources among a large set of potential source environments. With the unprecedented expansion of microbiome data repositories, such as the Earth Microbiome Project, recording over 200,000 samples from more than 50 types of categorized environments, STENSL takes the first steps in performing automated source exploration and selection. Using simulations, we found that STENSL is significantly more accurate in identifying the contributing sources as well as the unknown source, even when considering hundreds of candidate sources; settings in which state-of-the-art microbial source tracking methods add considerable error.

The utility of STENSL is established using two real data sets. The first is an *in vitro* data set we generated, in which we mixed six microbial environments, samples from the gut of humans and mice, and generated over two dozen sink samples. As this data set provides the ground truth rather than a simulation, we validated, for the first time, the generative model of common microbial source tracking methods (i.e., a sink is a convex combination of known and unknown sources). Next, we demonstrated the added value of STENSL, by showing it is robust to the presence of nuisance sources (i.e., sources that didn’t contribute to the formation of the sink). The second data set is the Human Microbiome Project. In this analysis, we showed STENSL’s ability to accurately identify the latent contributing sources, even in the presence of hundreds of nuisance ones, while significantly reducing estimation error.

Overall, using simulated and real sequencing data, we demonstrated that STENSL significantly improves the accuracy of microbial source tracking analysis over comparable methods by minimizing the contribution of nuisance sources and highlighting the actual contributing ones. By performing source selection that is robust to the presence of hundreds of nuisance sources, STENSL enables efficient source exploration using publicly available repositories thereby augmenting microbial source tracking analysis.

## MATERIALS AND METHODS

### The STENSL model.

Consider a single sink sample represented by a vector x where $x_{j}$ corresponds to the abundance of taxa j, 1  ≤  j  ≤  N. We define our model over M sources among which only a few sources may truly contribute to the sink, and therefore refer to them as candidates. Each source is represented by a vector $Y_{i}$, where $y_{\mathrm{ij}}$ is the observed abundance of taxa j in source i (1  ≤  i  ≤  M). Additionally, we assume there is an unobserved source (denoted as source M + 1). Let $C_{i}=\overset{N}{\underset{j=1}{\sum }}y_{\mathrm{ij}}$ and $C=\overset{N}{\underset{j=1}{\sum }}X_{j}$ be the total taxa counts of the candidate sources and sink respectively. With this notation, the generative model is as follows: we assume that there are mixture proportions—a vector α of length M + 1—where $\alpha_{i}$ corresponds to the fraction of source *i* in the sink, hence $\overset{M+1}{\underset{i=1}{\sum }}\alpha_{i}=1$. Thus far, the proposed model is similar to previous methods of estimating microbial mixtures by modeling it as a convex combination of sources (1). However, this model is limited when the number of sources is very large. To address such scenarios, we introduce the assumption that the vector α is sparse. Formally, we assume that the fraction of each source $\alpha_{i}$ follows an exponential distribution with the hyperparameter λ. In keeping α a valid mixing proportion from 1…M + 1, we work with the constraints that $\overset{M}{\underset{i=1}{\sum }}\alpha_{i}\leq 1$. The hyperparameter λ represents the level of sparsity in the contribution from the observed sources in our model. We also assume that the underlying relative abundance for each of the sources is unobserved, and that $Y_{i}$ are noisy realizations of these relative abundances. Formally, for each source i, we have a vector $\gamma_{i}$, where $\overset{N}{\underset{j=1}{\sum }}\gamma_{\mathrm{ij}}=\;1$. Each $\gamma_{\mathrm{ij}}$ represents the true relative abundance of taxa j in source i. Thus, the complete generative model for STENSL is given by:

$$\beta_{j}=\overset{M+1}{\underset{i=1}{\sum }}\alpha_{i}\gamma_{\mathrm{ij}}$$

$$Y_{i}\sim \mathrm{Multinomial}[C_{i},\left(\gamma_{i1},\ldots \gamma_{\mathrm{iN}}\right)],i\in [M]$$

$$Y_{M+1}\sim \mathrm{Multinomial}[C_{M+1},\left(\gamma_{M+1,1},\ldots \gamma_{M+1,N}\right)]$$

$$x\sim \mathrm{Multinomial}[C,\left(\beta_{1},\ldots \beta_{N}\right)]$$

$$\alpha_{1\ldots M}\sim \mathrm{Exp}(\lambda )\;1\{\overset{M}{\underset{i=1}{\sum }}\alpha_{i}\leq 1\}$$

We use an indicator function $1\{\overset{M}{\underset{i=1}{\sum }}\alpha_{i}\leq 1\}$ which is 1 when the condition parameter is true and 0 otherwise, such that we constrain the exponential distribution in a valid range. As we do not observe $Y_{M+1}$, we use a data-driven plug-in estimate ${\hat{Y}}_{M+1}$ as described below.

### Inference of STENSL parameters.

Under the STENSL model, we need to estimate the parameters α,γ,λ. Given the observed taxa counts in the candidate sources and sink, several inference algorithms could be used to estimate the parameters. For a fixed λ, we use expectation-maximization to infer parameters α and γ. We further introduce an initialization procedure according to the sparse model, as the initialization of the expectation-maximization (EM) algorithm (which attempts to optimize a nonconvex problem) is critical to the accuracy of the final estimates. For hyperparameter λ, we describe a grid-search algorithm to perform EM across a range of values, returning the set of parameters which obtains the highest likelihood as given by the generative model. The likelihood of the model is:

$$P(x,\;y_{1},\ldots ,y_{M}|\alpha ,\;\gamma ,\;\lambda )=(\begin{array}{c} \text{C}\\ x_{1},\ldots ,x_{N} \end{array})\;\overset{N}{\underset{j=1}{\prod }}{\left(\overset{M+1}{\underset{i=1}{\sum }}\alpha_{i}\gamma_{\mathrm{ij}}\right)}^{x_{j}}\;\overset{M}{\underset{i=1}{\prod }}[(\begin{array}{c} C_{i}\\ y_{i1},\ldots ,y_{iN} \end{array})\overset{N}{\underset{j=1}{\prod }}\gamma_{\mathrm{ij}}{}^{y_{\mathrm{ij}}}]\;\overset{M}{\underset{i=1}{\prod }}\lambda e^{-\lambda \alpha_{i}}1\{\overset{M}{\underset{i=1}{\sum }}\alpha_{i}\leq 1\}$$

The following log likelihood is defined under the constraint that $\overset{M}{\underset{i=1}{\sum }}\alpha_{i}\leq 1$:

$$l\;(\alpha ,\;\gamma ,\;\lambda )\;=\;\mathrm{logP}(x,\;y_{1},\ldots ,\;y_{M}|\alpha ,\;\gamma ,\;\lambda )\;=\overset{N}{\underset{j=1}{\sum }}x_{j}\;\log(\overset{M+1}{\underset{i=1}{\sum }}\alpha_{i}\gamma_{\mathrm{ij}})+\overset{M}{\underset{i=1}{\sum }}\overset{N}{\underset{j=1}{\sum }}y_{\mathrm{ij}}\;\log(\gamma_{\mathrm{ij}})-\overset{M}{\underset{i=1}{\sum }}\lambda \alpha_{i}+\mathrm{Mlog}\lambda +\mathrm{const}$$

Given λ which is assumed to be fixed in one instance of the EM algorithm, we obtain the expected complete log likelihood (Q) in terms of α and γ; we also introduce α^(*t*)^ and γ^(*t*)^ which are the parameters estimated in the previous iteration of the EM.

$$Q(\alpha ,\;\gamma ;\;\alpha^{\left(t\right)},\;\gamma^{\left(t\right)})\;=E[\mathrm{logP}]=\overset{M+1}{\underset{i=1}{\sum }}\overset{N}{\underset{j=1}{\sum }}x_{j}\;p(i\;|\;j)\;\log(\alpha_{i}\gamma_{\mathrm{ij}})+\overset{M}{\underset{i=1}{\sum }}\overset{N}{\underset{j=1}{\sum }}y_{\mathrm{ij}}\;\log(\gamma_{\mathrm{ij}})-\lambda \overset{M}{\underset{i=1}{\sum }}\alpha_{i}+\mathrm{Mlog}(\lambda )$$

where

$$\text{p}\left(\text{i }|\text{ j}\right)\;=\alpha_{i}{}^{\left(t\right)}\gamma_{\mathrm{ij}}{}^{\left(t\right)}/\overset{M+1}{\underset{i=1}{\sum }}\gamma_{\mathrm{ij}}{}^{\left(t\right)}\alpha_{i}{}^{\left(t\right)}$$

To complete the EM, we then maximize the E[LL] with respect to the parameters α and γ. We derive the maximization objective for α, for which λ appears as a sparsity hyperparameter and p(i | j) was obtained in the expectation step.

$$\alpha^{\left(t+1\right)}=\mathrm{argma}x_{\alpha }\;\overset{M+1}{\underset{i=1}{\sum }}\overset{N}{\underset{j=1}{\sum }}x_{j}\;p(i\;|\;j)\;\log(\alpha_{i})-\lambda \;||\;\alpha_{1:M}\;|{|}_{1}$$

s.t.

$$\overset{M}{\underset{i=1}{\sum }}\alpha_{i}\leq 1,\;\alpha \geq 0$$

Maximization for $\gamma_{\mathrm{ij}}$:

$$\gamma_{\mathrm{ij}}{}^{\left(t+1\right)}=\frac{x_{j}\;p(i\;|\;j)\;+\;y_{\mathrm{ij}}}{\sum_{j=1}^{N}x_{j}p(i\;|\;j)\;+\;y_{\mathrm{ij}}}$$

In summary, STENSL obtains the locally best solution for α and γ for given hyperparameter λ. We then test for increasing values of λ, the solution which reaches the highest likelihood under the model. The following pseudocode describes the steps of the STENSL algorithm.

STENSL Algorithm


Inputs: $\hat{\alpha },\hat{\gamma },{\hat{Y}}_{M+1}$ (initializations) and $\lambda_{1}<\ldots <\lambda_{K}$



Outputs: $\alpha_{\max},\gamma_{\max},\lambda_{\max}$



Repeat steps below for k=1… K



1. Initialize the EM problem with $\hat{\alpha },\hat{\gamma },{\hat{Y}}_{M+1}$. Fix $\lambda =\lambda_{k}$ as the given value for this problem



2. Perform EM to infer $\alpha_{k}$ and $\gamma_{k}$



3. $Q_{k}$ is the final likelihood obtained for the k-th problem



From $Q_{1}\ldots Q_{K}$, return $\alpha_{k},\gamma_{k},\lambda_{k}$, which obtained the highest likelihood


**Source selection.** STENSL assumes that only a sparse subset of the candidate sources contributes to the formation of the sink. To obtain a sparse selection of sources, we define a heuristic approach to infer the mixing proportions α under a sparsity assumption. In practice, inferring the latent taxa variables depend heavily on the observed taxa counts and motivates a heuristic that uses the observed counts directly to obtain an approximation of the hidden variables, with an added benefit that the approximation leads to an optimization problem that can be efficiently solved for large numbers of sources. A convenient choice for the initial values of γ are the observations $Y_{\mathrm{ij}}/\overset{N}{\underset{j=1}{\sum }}Y_{\mathrm{ij}}$, and we find that the observations are a sufficient proxy of $\gamma_{\mathrm{ij}}$ in approximating α. We next perform inference by formulating a least-squares problem between x and $Y^{T}\alpha$ while ensuring that α is sparsely approximated by L1 regularization. We specifically choose the L1 regularization (Lasso), which follows this formulation, with added constraints that each proportion must be nonnegative and $\overset{M}{\underset{i=1}{\sum }}\alpha_{i}\leq 1$. In leveraging Lasso, we assume the presence of underlying noise which is normally distributed when forming the sink. We leverage this underlying noise to estimate the abundance profile of the unknown source. We therefore seek to optimize the following objective function, where we determine an optimal value for hyperparameter λ through cross-validation (Fig. S3):

$$\hat{\alpha }\;=\mathrm{argmin}\;||\;Y^{T}\alpha -X\;|{|}_{2}^{2}+\lambda \;||\;\alpha \;|{|}_{1}$$

$$\mathrm{subject}\;\mathrm{to}\;\overset{M}{\underset{i=1}{\sum }}\alpha_{i}\leq 1,\;\alpha_{i}\geq 0$$

10.1128/mSystems.00995-21.3FIG S3Cross-validation to determine λ. STENSL utilizes Lasso as part of the selection step, in which hyperparameter **λ** of the sparsity regularization must be determined. We use cross-validation on a simulated dataset of sinks (and their corresponding sources) to find an optimal **λ** for microbial data based on prediction errors $\epsilon =\frac{1}{M}\overset{M}{\underset{i=1}{\sum }}{\left(\text{X}-Y^{T}\alpha \right)}^{2}$. The dataset is split in half for sinks which will be analyzed as part of training to tune the hyperparameter, and we evaluate Lasso on the reserved test set. In the validation plot, we show the average of the errors ϵ for each **λ** and find that choosing a value < 1 × 10^−4^ would be optimal. In our experiments, we chose λ = 1 × 10^−6^. Download FIG S3, TIF file, 0.1 MB.Copyright © 2022 An et al.2022An et al.https://creativecommons.org/licenses/by/4.0/This content is distributed under the terms of the Creative Commons Attribution 4.0 International license.

The objective describes a least-squares problem which is solvable as a quadratic program with the stated constraints. From the initial sparse approximation of α, we consider candidate source *i*, for which contribution is positive $\alpha_{i\;}>0$, to be highly likely to be contributing to the sink. We use the sparsely estimated proportions α as initialization values, denoted as $\hat{\alpha }$, in proceeding with expectation-maximization according to our model.

**Unknown source initialization.** In obtaining an initial estimate of the unknown abundances counts $Y_{M+1}$, we assume that its underlying relative abundance follows a truncated normal distribution similar to the nonnegative components of the estimation noise resulting from the selection step. In practice, to remove the prominent signal stemming from the true known sources, we subtract the scaled counts of the top contributing source, ranked based on the L-1 regularization and thus, ${\hat{Y}}_{M+1}=\max(0,x-\hat{\alpha }Y_{\mathrm{argma}x_{}\left(\hat{\alpha }\right)})$.

### Metrics.

We measured the accuracy of the estimated source contributions in terms of overall error with respect to the ground truth, the identification of, specifically, the unknown proportion, and the proportion of falsely identified contributions which we termed “false positive rate.” Overall error of the mixing proportion estimated for M sources $\hat{\alpha }$ was measured against the simulated or ground truth proportions α using mean-squared error $\mathrm{MSE}=\overset{M}{\underset{i=1}{\sum }}{\left(\alpha_{i}-{\hat{\alpha }}_{i}\right)}^{2}$. In evaluating the unknown proportion identified in the sink, we used absolute error $\mathrm{AE}\;=\;|\;\alpha_{M+1}-{\hat{\alpha }}_{M+1}\;|$. The false-positive rate referring to positive proportions incorrectly attributed to known noncontributing sources was calculated as the sum of such proportions, $\mathrm{FPR}=\overset{M}{\underset{i=1}{\sum }}1_{\alpha_{i}=0}\;{\hat{\alpha }}_{i}$.

### Simulation procedure.

To examine the accuracy of STENSL, we used multiple source environments with varying degrees of overlap in their distribution by randomly sampling from the Earth Microbiome Project. Each source environment was subsampled to contain 10,000 reads. In each iteration of the simulation, we sampled M + 1 candidate environments and used them to build a synthetic sink with different mixing proportions. To simulate an unknown source as well as sparsity in source contribution, only K source environments were designated as contributing sources. We used 30 mixing proportions (corresponding to 30 simulated sinks) and K = 10 contributing sources in each iteration with M = 50. We drew the mixing vector of length K from a Pareto distribution, which was scaled to sum to 1 for mixtures with no unknown. To simulate sinks with unknown presence, an unknown proportion of up to 90% was introduced by scaling the drawn vector to ≤1, then appending the unknown proportion. Finally, the sink was generated under the model as a linear combination of the K contributing sources and the unknown source. For a detailed description of the simulation, see Supplementary Material.

### *In vitro* data generation.

To evaluate the performance of STENSL and validate the mixture model assumed by common microbial source tracking methods, we generated *in vitro* data, using the generative model described above following a 16S amplification protocol from Tong et al. (10). The contributing sources were taken from the digestive systems of three human subjects and three mice. Two of the human subjects were documented with a pre-Ketogenic diet and the third was sampled from the Human Altitude Study. Using these sources, we assembled 27 *in vitro* sinks, each sink composed of two to three microbial samples at varying mixing proportions (ranging from 20%–80%). For a detailed description of the assembly process and protocols used, see Supplementary Material. To assess the performance of microbial source tracking methods in the presence of noncontributing sources, we next generated 50 additional synthetic sources by shuffling the abundances of the six contributing sources described above. The number of taxa expressed in each synthetic source $T_{i}$; (1 ≤ i ≤ 50) was determined following a uniform distribution $T_{i}\sim \mathrm{Unif}[\min\left(T_{\mathrm{rea}l_{1}},\ldots ,T_{\mathrm{rea}l_{6}}\right),\max\left(T_{\mathrm{rea}l_{1}},\ldots ,T_{\mathrm{rea}l_{6}}\right)]$, where $T_{\mathrm{rea}l_{j}};\;(1\leq j\leq 6)$ is the number of taxa in the six contributing sources described above. Then, for each taxon-j, we randomly drew, without replacement, a count which was observed among all nonzero taxa.

### Data availability.

The code for STENSL is a branch of the FEAST codebase, which can be found on https://github.com/cozygene/FEAST/tree/STENSL. We also created a short tutorial of STENSL that can be found on https://github.com/cozygene/FEAST/blob/STENSL/vignettes/STENSL_example.R.

10.1128/mSystems.00995-21.8 TEXT S1 Description of the in-silico simulations. Download Text S1, DOCX file, 0.01 MB. Copyright © 2022 An et al. 2022 An et al. https://creativecommons.org/licenses/by/4.0/ This content is distributed under the terms of the Creative Commons Attribution 4.0 International license.

10.1128/mSystems.00995-21.9 TEXT S2 Description of in-vitro data generation. Download Text S2, DOCX file, 0.01 MB. Copyright © 2022 An et al. 2022 An et al. https://creativecommons.org/licenses/by/4.0/ This content is distributed under the terms of the Creative Commons Attribution 4.0 International license.

10.1128/mSystems.00995-21.10 TEXT S3 Description of Earth Microbiome Project experiments. Download Text S3, DOCX file, 0.01 MB. Copyright © 2022 An et al. 2022 An et al. https://creativecommons.org/licenses/by/4.0/ This content is distributed under the terms of the Creative Commons Attribution 4.0 International license.
