# Supplementary material for: STENSL: Microbial Source Tracking with ENvironment SeLection
Source: mSystems. 2022 Sep 1;7(5):e00995-21. doi: 10.1128/msystems.00995-21 (PMC9599664; doi:10.1128/msystems.00995-21)
Supplement: TABLE S1 [file msystems.00995-21-s0007.docx]

1. Difference in means, MSE

| Unknown | 0% | 20% | 40% | 70% | 90% |
| --- | --- | --- | --- | --- | --- |
| FEAST | Not sig. | Not sig. | p=5.40e-24 | p=5.59e-31 | p=1.03e-19 |
| STracker2 | Not sig. | p=0.0397 | p=8.39e-17 | p=1.01e-34 | p=4.87e-26 |
| RAD | Not sig. | p=7.47e-4 | p=3.93e-18 | p=4.39e-35 | p=5.29e-27 |

1. Difference in means, noise amount

| Unknown | 0% | 20% | 40% | 70% | 90% |
| --- | --- | --- | --- | --- | --- |
| FEAST | p=0.0361 | p=2.84e-11 | p=1.39e-23 | p=4.86e-26 | p=2.69e-18 |
| STracker2 | p=5.96e-13 | p=4.67e-23 | p=3.63e-24 | p=6,86e-27 | p=3.41e-22 |
| RAD | p=2.13e-31 | p=2.37e-29 | p=6.48e-27 | p=9.61e-26 | p=5.37e-13 |

(c) Difference in means in unknown estimation, absolute error

| Unknown | 0% | 20% | 40% | 70% | 90% |
| --- | --- | --- | --- | --- | --- |
| FEAST | Not Sig. | p=2.24e-18 | p=2.40e-37 | p=9.50e-29 | p=1.38e-19 |
| STracker2 | p=1.07e-4 | p=1.36e-20 | p=1.52e-41 | p=3.11e-34 | p=6.94e-26 |
| RAD | Not sig. | p=2.61e-21 | p=2.88e-44 | p=8.77e-36 | p=6.98e-27 |
