# Supplementary figures and images for: STENSL: Microbial Source Tracking with ENvironment SeLection
Source: mSystems. 2022 Sep 1;7(5):e00995-21. doi: 10.1128/msystems.00995-21 (PMC9599664; doi:10.1128/msystems.00995-21)

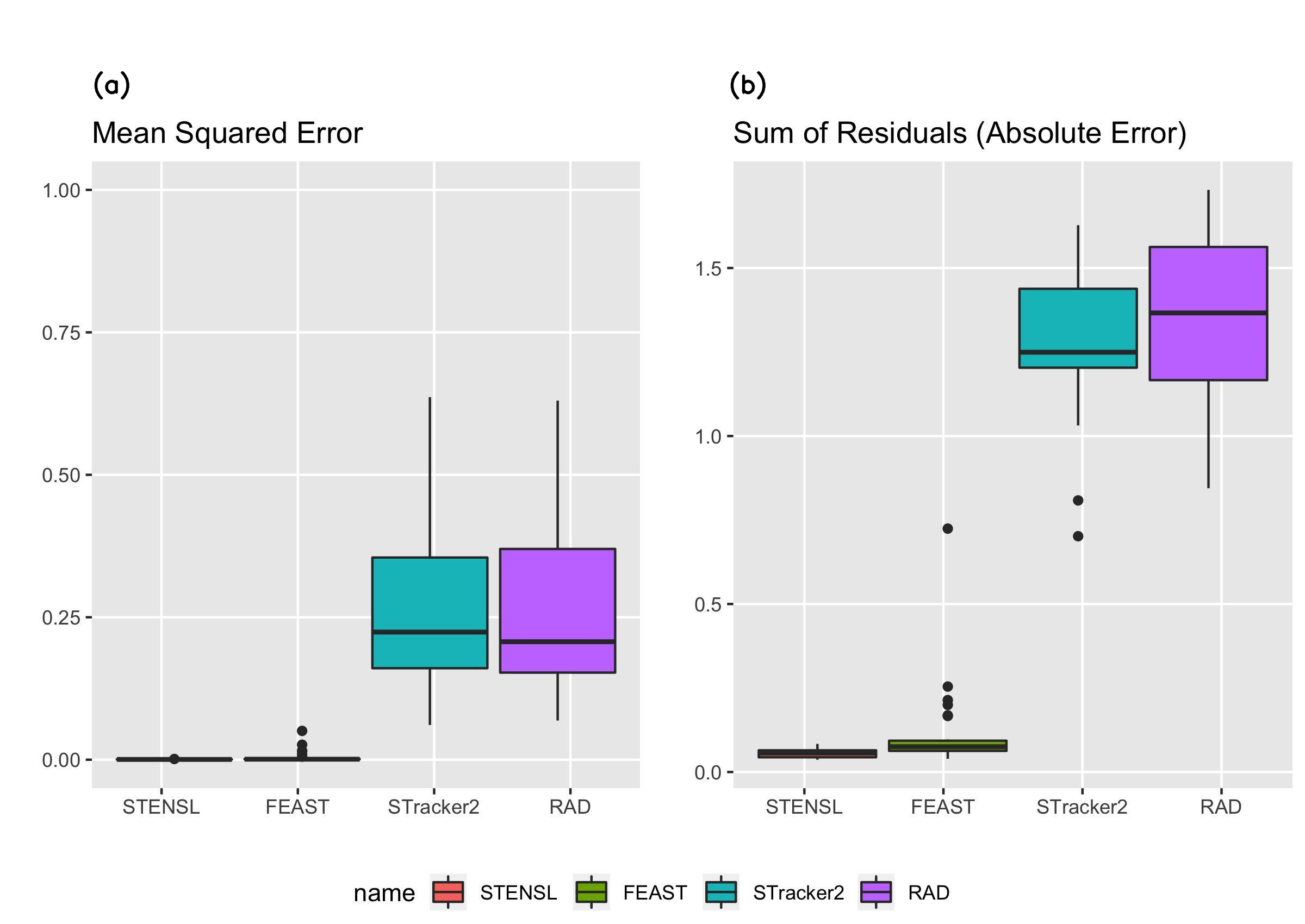

Supplement: FIG S1 [file msystems.00995-21-s0001.tif]

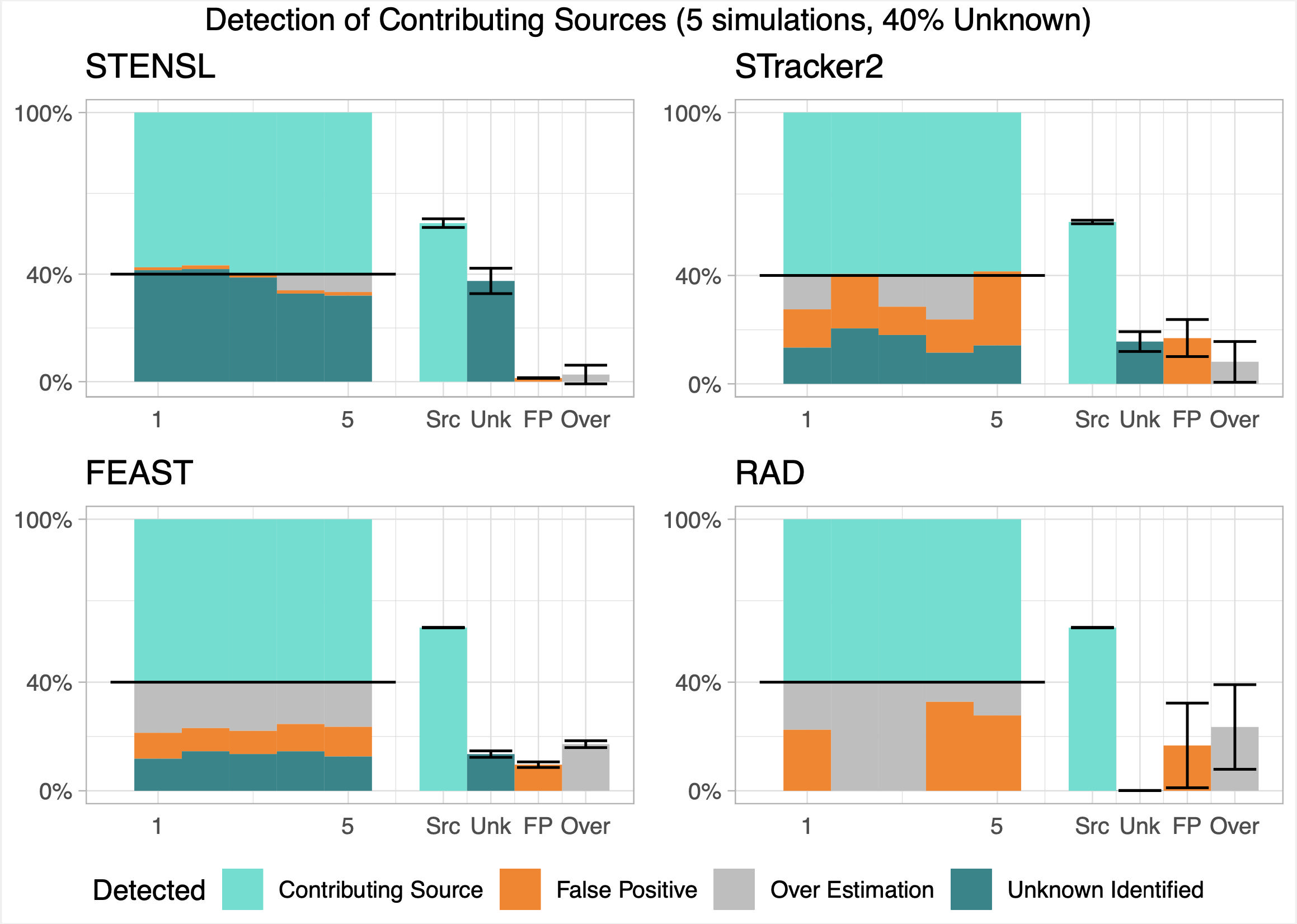

Supplement: FIG S2 [file msystems.00995-21-s0002.tif]

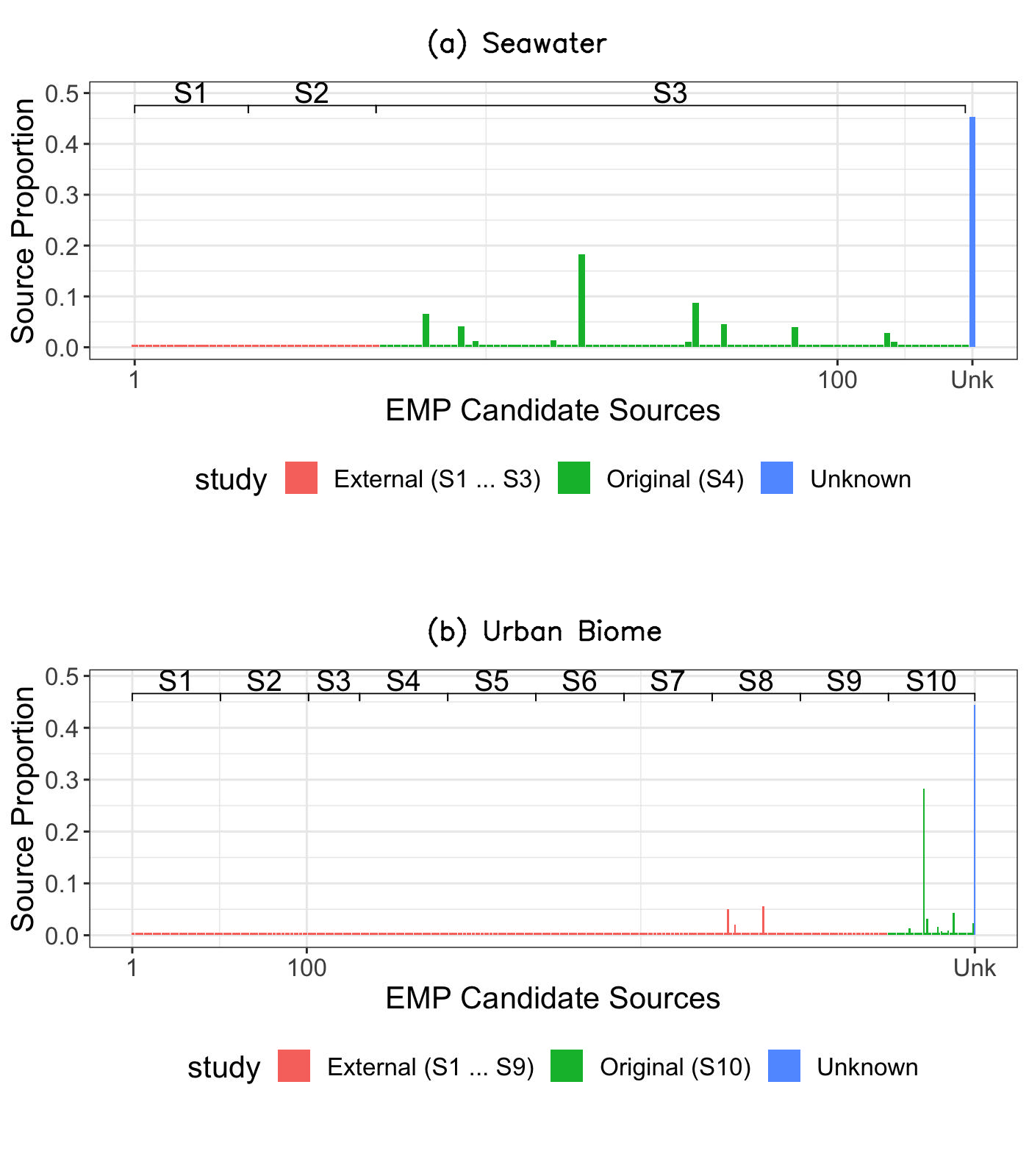

Supplement: FIG S6 [file msystems.00995-21-s0006.tif]

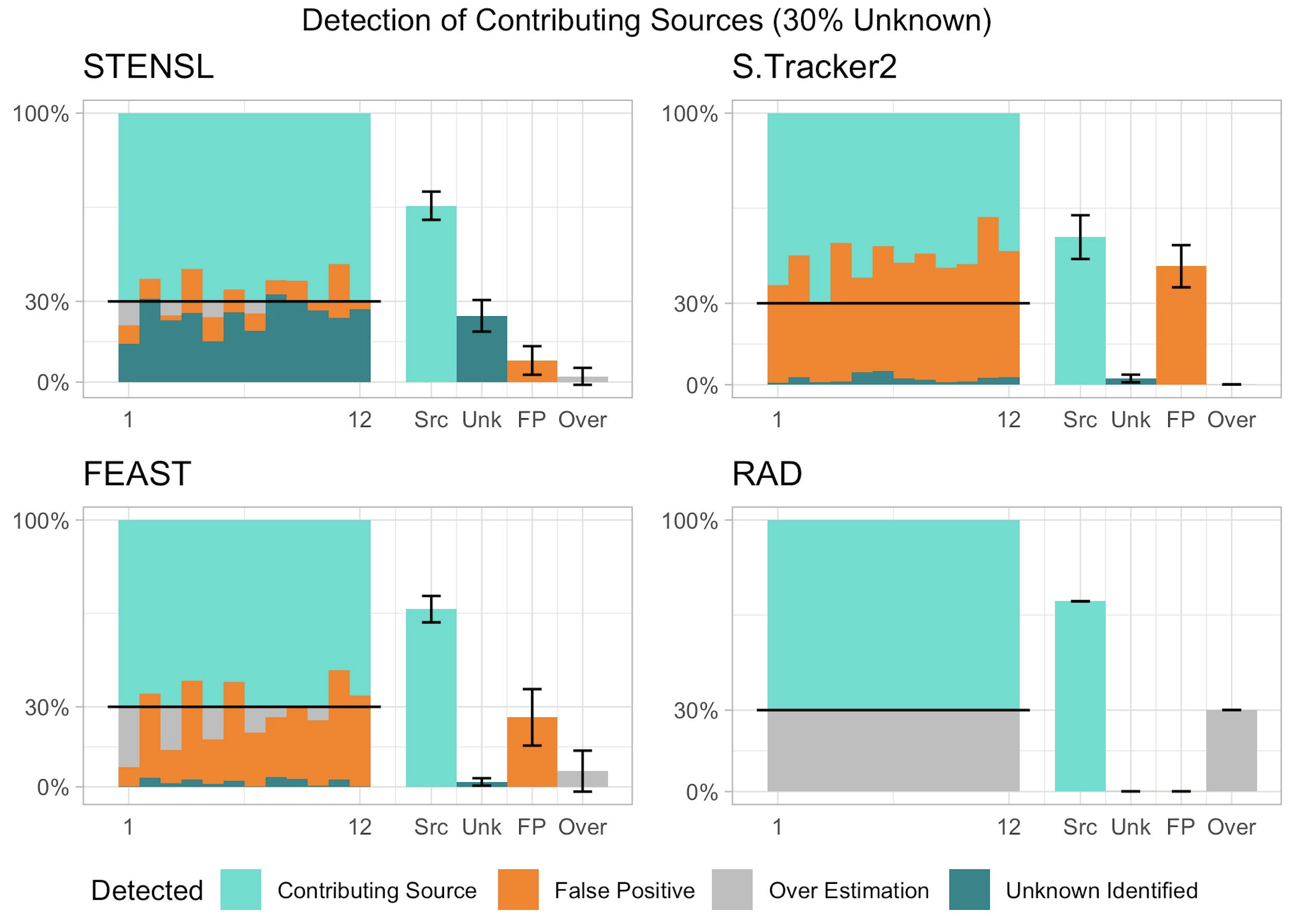

Supplement: FIG S5 [file msystems.00995-21-s0005.tif]

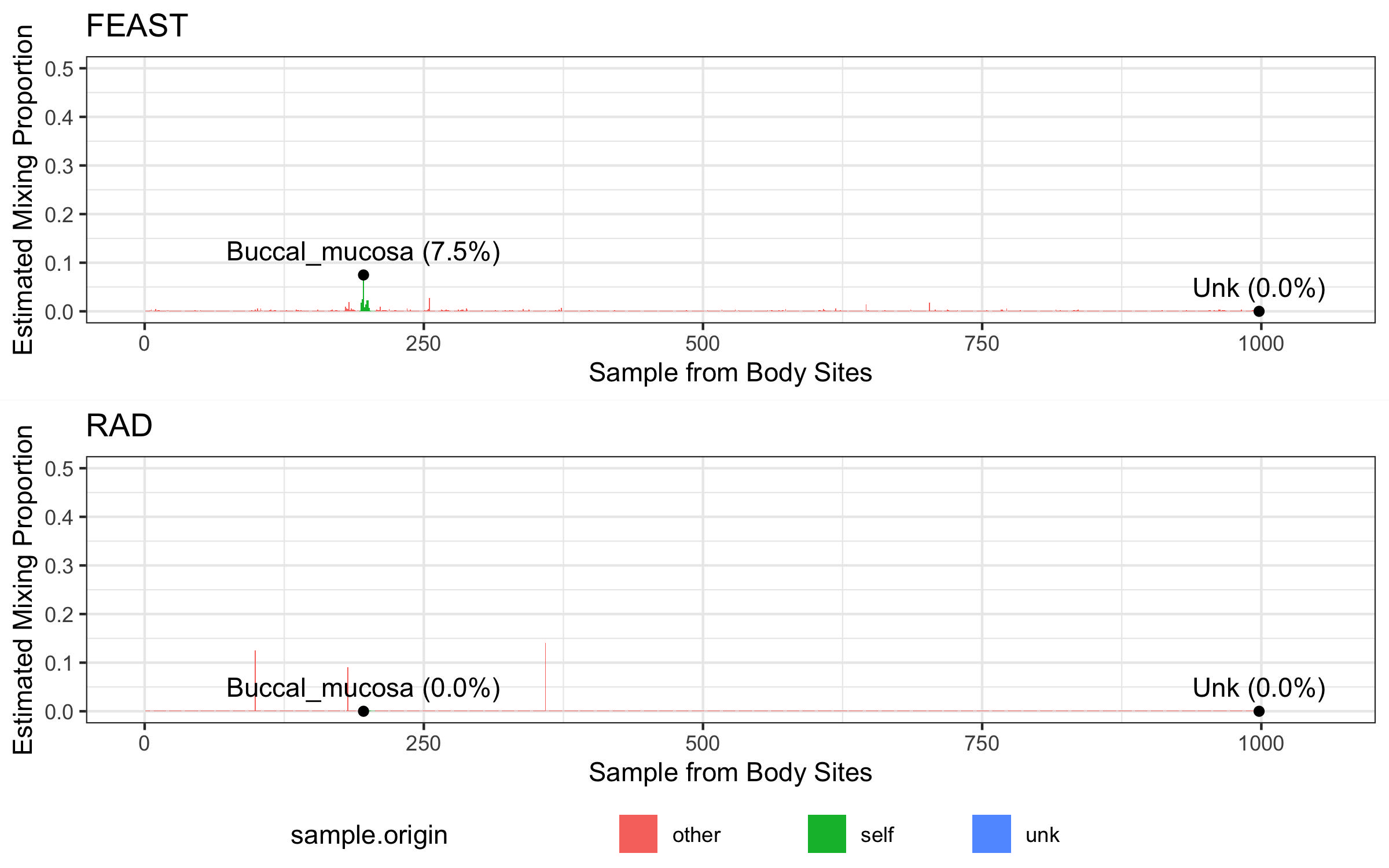

Supplement: FIG S4 [file msystems.00995-21-s0004.tif]

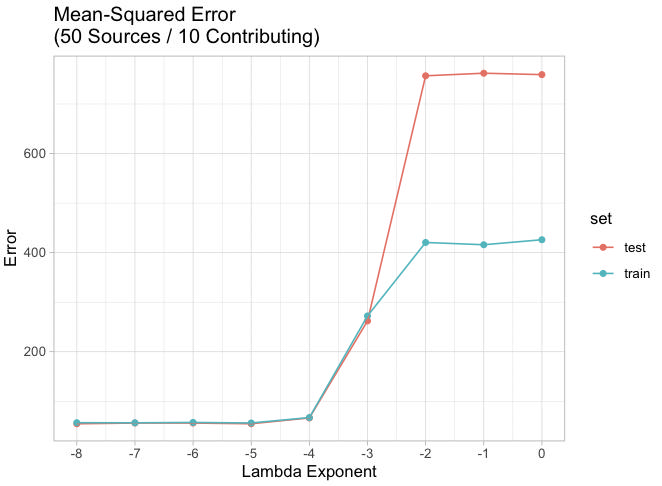

Supplement: FIG S3 [file msystems.00995-21-s0003.tif]
