# Supplementary material for: STENSL: Microbial Source Tracking with ENvironment SeLection
Source: mSystems. 2022 Sep 1;7(5):e00995-21. doi: 10.1128/msystems.00995-21 (PMC9599664; doi:10.1128/msystems.00995-21)
Supplement: TEXT S1 [file msystems.00995-21-s0008.docx]

**Supplementary Note 1**

**Simulation procedure.** We evaluated STENSL under a variety of sinks generated using real-world microbial samples found in the Earth Microbiome Project^8^. In practice, we may encounter minimal to dominant unknown presence, so we simulated sinks for unknown percent from 0%~90%. We denote the unknown proportion according to the percentages as $u$. In addition, we ensured that the sources were sufficiently distinct from each other by thresholding pairwise Jensen-Shannon Divergence (JSD). As many as M sources were gathered per sink, of which K sources randomly contributed to the sink. Accuracy for the generated sinks was measured as the Mean-Squared Error (MSE) between the estimated and true mixing proportions.

We used the following simulation procedure to generate a single sink for chosen unknown proportion $u\in\{0.2, 0.4, 0.6, 0.7, 0.9\}$:

1. Choose number of sources M and K, where $K\leq M$
2. Randomly generate a mixing proportion for K sources such that $\bar{m_{1}}...\bar{m}_{K}\sim Pareto(\alpha>0).$Normalize the proportions such that $\boldsymbol{m}_{\boldsymbol{k}}\boldsymbol{=}\frac{\boldsymbol{1-u}}{\sum_{\boldsymbol{k=1}}^{\boldsymbol{K}} \boldsymbol{m}_{\boldsymbol{k}}}{\bar{\boldsymbol{m}}}_{\boldsymbol{k}}$ where $u$ is reserved for the unknown proportion.
3. Draw M microbial samples $S_{1}, ... , S_{M}$ from the dataset. We ensure pairwise divergence $JSD(S_{i}, S_{j}) > 0.8$ for $1\leq i\leq M, 1\leq j\leq M$.
4. Draw the M+1^th^ microbial sample $S_{M+1}$ which is the unknown source. Ensure pairwise divergence $JSD(S_{i}, S_{M+1}) > 0.5$for $1\leq i\leq M$.
5. Draw noisy realizations of real sources $S_{1}, ... , S_{M+1}$from the Multinomial distribution which we denote as $Y_{1}, ... , Y_{M+1}$.
6. The sink sample abundance is obtained as $\sum_{k\in\{1...K, M+1\}} m_{k}S_{k}=X$. This combines the first K randomly selected sources and the unknown source.
7. STENSL is applied to sink X and sources $Y_{1}, ... , Y_{M+1}$.
8. Calculate the MSE between the estimated and true mixing proportions.
