# Supplementary material for: STENSL: Microbial Source Tracking with ENvironment SeLection
Source: mSystems. 2022 Sep 1;7(5):e00995-21. doi: 10.1128/msystems.00995-21 (PMC9599664; doi:10.1128/msystems.00995-21)
Supplement: TEXT S2 [file msystems.00995-21-s0009.docx]

**Supplementary Note 2**

**In vitro data generation.** The following materials were used: (a) 96 well plate of uniquely barcoded IL_806r primers[^11^](https://paperpile.com/c/Oooo8i/aNDJ), (b) ILHS_515f, (c) 10 $\times$ PCR buffer (Sigma-Aldrich, cat. no. D9307) 10 mM dNTPs JumpStart Taq DNA polymerase (Sigma-Aldrich, cat. no. D9307), (d) DEPC-treated water QIAquick PCR purification kit (QIAGEN, cat. no. 28104), (e) Nanodrop spectrophotometer, (f) Thermal cycler, (g) six microbial environments: (1) Mouse SPF after 7 days of Ketogenic Diet, (2) Mouse SPF with after 7 days of standard diet, (3) Mouse SPF Kcna +/+ 1, (4) human subject 1, Pre-Ketogenic diet, (5) human subject 2, from the 'Human Altitude Study', SA2 and (6) human subject 3, Pre-Ketogenic diet. Overall, 27 synthetic sinks were assembled in vitro with varying source contributions, using two to three microbial environments per sink. We Prepared the following PCR master mix to amplify each sample in triplicate: 10× PCR buffer 3$\mu l$ x 3.3 x no. of samples dNTPs (10 mM) 0.6$\mu l$ x3.3 x no. of samples JumpStart Taq DNA polymerase 0.3$\mu l$ x 3.3 x no. of samples H2O 23.1$\mu l$ x 3.3 x no. of samples. For this step, each separate source used in a sample was amplified separately, then purified via PCR purification, then added to the final multiplexed tube in a proportion according to the outlined above so that the total amount added is 250 ng of DNA per barcode. This is to prevent sequencing bias. i.e., for Barcode number 1, if this is a sample prepared as 1a, this would mean sample number 1 and number 2 would be amplified in separate tubes using Barcode 1, then 125 ng of purified product will be used for each in the final tube (to get 50% of each).

We added 81$\mu l$ master mix to every third well of a 96-well PCR plate, added 6$\mu l$ sample DNA (2$\mu l$ x 3 reactions) and 3$\mu l$ primer mix (1$\mu l$ x 3 reactions) from a well of a 96-well primer plate to each of these wells. Using a P200 pipette, we mixed each well and then transferred 30$\mu l$ into each of the two neighboring wells, resulting in triplicate reactions for each sample. we Sealed the plate with 8-cap strips (made sure they are matched to the plate) or with plastic film. We performed PCR with the following thermal cycler settings: 1 cycle: 94 degree Celsius 3 min 35 cycles: 94 degrees Celsius 45 sec 50 degrees Celsius 1 min 72 degrees 1.5 min 1 cycle: 72 degrees Celsius 10 min 1 cycle: 72 degrees Celsius 5 min Final step: 4 degrees Celsius (hold). We combined the PCR products from the triplicate reactions. We used the Qiagen PCR cleanup kit to purify PCR products from each sample and elute DNA with 30$\mu l$ PCR grade (DEPC-treated) H2O. We quantified the amplified DNA using a spectrophotometer, and then combined 250 ng of each sample amplicon into one tube to make a multiplexed library. Samples were multiplexed using the appropriate sequencing primers[^11^](https://paperpile.com/c/Oooo8i/aNDJ), to Laragen for sequencing. We obtained ~70,000 reads per sample. All samples were analyzed via QIIME2 using ASV and taxonomic information[^12^](https://paperpile.com/c/Oooo8i/NEnT).
