# Supplementary material for: STENSL: Microbial Source Tracking with ENvironment SeLection
Source: mSystems. 2022 Sep 1;7(5):e00995-21. doi: 10.1128/msystems.00995-21 (PMC9599664; doi:10.1128/msystems.00995-21)
Supplement: TEXT S3 [file msystems.00995-21-s0010.docx]

**Supplementary Note 3**

**Earth Microbiome Project (EMP) Multi-Study Experiment** We performed several experiments on combinations of studies from the EMP to determine if STENSL was effective in identifying reasonable sources. We combined up to 10 studies which were related (e.g. by sampling environment such as seawater or by biome such as urban) but varying in the locations or time of the studies. Then, in each experiment we randomly chose 1 sample to treat as a sink. The sink's origin could be traced back to a specific study, but no such prior information was given to the algorithm. Then, we performed source estimation using STENSL to examine which of the sources spanning multiple studies would be detected. We observed that STENSL was capable of distinguishing source samples belonging to the original study and attributing minimal proportion to all other samples which were considered from other similar studies (Figure S6). We also observed that STENSL could identify a non-zero amount of unknown proportion where the sink could not be fully explained, instead of distributing the proportion across many unrelated sources. Therefore, we verified that STENSL could handle a high number of sources which can be found in the EMP.
